# Translation, cultural adaptation, and psychometric validation of the Arabic version of the Digital Life Balance Scale in an Arabic-speaking university student sample

**DOI:** 10.3389/fpsyg.2026.1741166

**Published:** 2026-02-12

**Authors:** Aamer Aldbyani, Afnan Alhimaidi

**Affiliations:** 1 Shandong Xiehe University, Jinan, China; 2 Princess Nourah bint Abdulrahman University, Riyadh, Saudi Arabia

**Keywords:** Arabic version, digital life balance, reliability, university students, validity

## Abstract

**Introduction:**

The increasing integration of digital technologies into daily life has underscored the need for valid instruments to assess individuals’ ability to maintain a balanced engagement between online and offline activities within Arabic-speaking university student samples. This study aimed to evaluate the validity and reliability of the Arabic version of the Digital Life Balance (DLB) Scale among Arab university students.

**Methods:**

A total of 769 students participated (Saudi Arabia = 261; Yemen = 311; Egypt = 197). The scale was translated into Arabic, and its linguistic and cultural equivalence was established through expert evaluation using the Delphi technique. Convergent validity was examined using three subscales of the Arabic Digital Stress Scale (DSS-A)—Fear of Missing Out, Connection Overload, and Online Vigilance.

**Results:**

Confirmatory factor analysis supported the one-factor structure of the DLB Scale in the pooled Arabic-speaking sample, indicating clear construct validity. The DLB Scale demonstrated strong internal consistency (Cronbach’s α = .87; McDonald’s ω = .80; Composite Reliability = .89). Small but significant negative correlations were found between DLB scores and Fear of Missing Out (*r* = −.10, *p* = .006) and Connection Overload (*r* = −.15, *p* < .001), whereas the association with Online Vigilance was negative but not statistically significant (*r* = −.07, *p* = .060).

**Discussion:**

Taken together, these findings provide evidence for the reliability and factor structure of the Arabic version of the DLB Scale, with partial support for convergent validity, within the studied Arabic-speaking university student sample.

## Introduction

The rapid integration of digital technologies into daily routines has transformed how individuals manage their time, social interactions, and overall well-being. This transformation has drawn increasing attention to the concept of Digital Life Balance (DLB), which refers to an individual’s capacity to integrate digital engagement into everyday life in ways that support—rather than compromise—psychological, social, and physical health (Duradoni et al., 2022). DLB emphasizes the adaptive regulation of technology use, enabling individuals to maintain equilibrium between online and offline domains.

Recent research has conceptualized DLB as a multidimensional construct that encompasses behavioral, cognitive, and emotional aspects of adaptive digital engagement (Duradoni et al., 2024; Chen et al., 2025). Empirical studies have shown that higher levels of DLB are associated with lower levels of technology-related stress, greater self-regulation, and improved social functioning (Aldbyani et al., 2025a; Duradoni et al., 2024; Malas et al., 2025). Collectively, these findings indicate that DLB is associated with indicators of psychological adjustment and overall well-being within increasingly digitalized environments. Moreover, recent findings emphasize the role of mindfulness and self-regulation in maintaining digital balance and reducing problematic smartphone use (e.g., Aldbyani et al., 2025a, 2025b; Throuvala et al., 2020; Wadhwa and Satpathy, 2020).

The rapid diffusion of digital tools has blurred traditional boundaries between professional and personal life, creating new challenges for achieving digital balance. Empirical reviews indicate that increased smartphone use for work during off-job hours is significantly associated with higher work–life conflict, decreased psychological detachment, and impaired well-being (Blake et al., 2024; van Zoonen et al., 2020). Thus, DLB is not limited to personal or leisure contexts but also reflects the ability to manage technology boundaries across occupational and domestic spheres.

Recent findings have highlighted that digital life balance is closely related to affective experiences such as Fear of Missing Out (FoMO), loneliness, and digital social capital. Individuals with higher DLB levels report lower FoMO and perceived isolation, together with greater online–offline social connectedness (Coyne and Woodruff, 2023). This evidence reinforces the notion that DLB supports not only cognitive self-regulation but also emotional stability and social well-being, emphasizing its multidimensional nature.

While much DLB research emphasizes psychological and social outcomes, there is emerging empirical evidence of its physical and behavioral implications. For example, intervention studies show that reducing smartphone use or increasing physical activity leads to improvements in mood, well-being, and digital self-regulation (Precht et al., 2024). Furthermore, systematic reviews link excessive screen use with sleep disturbances, metabolic dysregulation, and other physical health risks (Mazumdar, 2025). These findings broaden the conceptual scope of DLB, underscoring that maintaining digital balance involves behavioral and somatic regulation in addition to psychological adaptation.

Although both DLB and problematic technology use describe patterns of digital interaction, they represent distinct constructs. DLB reflects a balanced and purposeful approach to technology that enhances personal and social well-being, whereas problematic use is characterized by compulsive behaviors that interfere with daily functioning. Validation studies of the DLB Scale have consistently demonstrated discriminant validity when compared with measures of problematic smartphone or internet use within Arabic-speaking university student samples (Aldbyani et al., 2025a; Duradoni et al., 2022; Soysal et al., 2024). This distinction is theoretically important because DLB reflects adaptive regulation of digital engagement, whereas problematic technology use reflects maladaptive, compulsive patterns that interfere with daily functioning. Practically, clarifying this distinction ensures that DLB is not merely the opposite of problematic use but represents a positive indicator of balanced and regulated digital behavior, as supported by previously validated discriminant evidence.

Previous studies have examined the Digital Life Balance construct in multiple linguistic and cultural settings, supporting its theoretical relevance across different populations. Adaptations of the DLB Scale in Brazilian, Turkish, and other linguistic contexts have reported satisfactory psychometric properties within the examined samples (Lima-Costa et al., 2024; Soysal et al., 2024; Tosti et al., 2025). However, despite growing awareness of the importance of maintaining balanced digital engagement in Arab societies, no validated Arabic version of the DLB Scale currently exists. This research gap constrains both empirical investigation and applied assessment in the Arab region, especially amid the expanding digitalization of education, work, and everyday life.

Addressing this gap requires rigorous translation, cultural adaptation, and psychometric evaluation of the DLB Scale to ensure its validity and reliability for use in Arab populations. The present study aimed to translate and culturally adapt the Arabic version of the Digital Life Balance Scale (DLBS) and evaluate its psychometric properties among university students in Saudi Arabia, Yemen, and Egypt. Convergent validity was examined by assessing the associations between DLBS scores and three dimensions of the Arabic Digital Stress Scale—Fear of Missing Out, Connection Overload, and Online Vigilance. Establishing a reliable and valid Arabic version of the DLB Scale will provide researchers and practitioners with a standardized and culturally relevant tool for evaluating individuals’ perceived balance between their digital and offline lives within Arab contexts.

### The current study

The translation and cultural adaptation of psychological measurement instruments are critical for ensuring construct validity across cultural groups. Instruments developed in one linguistic or cultural context may not fully capture the intended psychological construct when applied elsewhere, potentially yielding biased or misleading results. In Arab contexts, there remains a notable shortage of standardized tools that assess emerging constructs such as Digital Life Balance (DLB). Given the accelerating integration of digital technologies into daily life, there is an urgent need for culturally adapted instruments capable of assessing individuals’ perceived ability to balance online and offline activities.

The original English version of the DLB Scale (Duradoni et al., 2022), composed of four items assessing perceived ability to integrate digital engagement into daily life in ways that support well-being, has been validated in several languages. However, a reliable Arabic version suitable for Arab populations has not yet been developed. Accordingly, systematic translation, cultural adaptation, and psychometric evaluation are necessary to establish the scale’s applicability and measurement equivalence in Arab settings.

The present study, therefore, aimed to translate, culturally adapt, and validate the Arabic version of the DLB Scale (DLBS) among Arabic-speaking university students recruited from Saudi Arabia, Yemen, and Egypt. To assess convergent validity, three theoretically relevant subscales from the Arabic version of the Digital Stress Scale (DSS-A)—Fear of Missing Out, Connection Overload, and Online Vigilance—were employed, as these dimensions represent distinct yet conceptually related aspects of digital stress expected to correlate negatively with DLB.

Based on these objectives, the following hypotheses were formulated:

*H1*: The Arabic version of the DLB Scale will demonstrate satisfactory linguistic and cultural equivalence with the original English version.

*H2*: The Arabic DLB Scale will exhibit adequate construct validity as supported by confirmatory factor analysis.

*H3*: The Arabic DLB Scale will demonstrate acceptable convergent validity, reflected by significant negative correlations with the three selected dimensions of the Digital Stress Scale (Fear of Missing Out, Connection Overload, and Online Vigilance).

*H4*: The Arabic DLB Scale will show satisfactory internal consistency and composite reliability.

## Methods

### Participants

The study sample comprised 769 students recruited from three Arab countries: Saudi Arabia (261), Yemen (311), and Egypt (197). The recruitment process followed a convenience sampling strategy and targeted Arabic-speaking students enrolled in accredited higher-education institutions. Although participants were drawn from multiple countries, the sampling procedure was not designed to generate balanced or representative national subsamples. Instead, the sample reflects a pooled Arabic-speaking population rather than a stratified multi-country design. Accordingly, the present study treats the sample as a pooled Arabic-speaking student population, while measurement invariance across countries was empirically examined rather than assumed.

Participants represented diverse academic levels, including diploma, bachelor’s, master’s, and doctoral programs. Participants’ mean age was 24.8 years (SD = 2.4). The overall sample included 366 males (47.6%) and 403 females (52.4%). Gender distribution varied by country, with female participants comprising approximately 75% of the Saudi subsample, 55% of the Egyptian subsample, and 32% of the Yemeni subsample. These country-level characteristics are reported descriptively to enhance transparency and are not intended to support cross-country comparisons.

### Instrument

#### Digital life balance

The Digital Life Balance Scale (DLBS) is a brief self-report instrument designed to assess individuals’ perceived ability to maintain a healthy balance between online and offline activities in daily life (Duradoni et al., 2022). The scale consists of four items, each rated on a 7-point Likert scale ranging from 1 (*strongly disagree*) to 7 (*strongly agree*), with higher scores indicating greater perceived balance between digital and non-digital domains. The Arabic version of the DLBS used in the present study was developed and validated according to established cross-cultural adaptation procedures (see the *Translation and Cultural Adaptation section*). In the current study, the DLBS demonstrated satisfactory internal consistency, with Cronbach’s α = 0.87 and McDonald’s *ω* = 0.80, supporting its reliability as a measure of digital life balance.

#### Digital stress scale

The Arabic version of the Digital Stress Scale (DSS-A) was used to assess participants’ perceived levels of digital stress. The instrument was originally developed in English by Hall et al. (2021) to measure five dimensions of digital stress: availability stress, approval anxiety, fear of missing out, connection overload, and online vigilance. For the present study, only the last three dimensions—fear of missing out (4 items), connection overload (5 items), and online vigilance (5 items)—were employed. These subscales were selected because they represent the domains most theoretically aligned with digital life balance, particularly aspects of digital overload, dysregulation, and socio-digital attentional strain. The remaining two subscales assess broader forms of digital tension that fall outside the focal scope of digital balance; therefore, their exclusion narrows the convergent validity assessment to the stress components most conceptually related to the DLBS construct. Each item was rated on a 5-point Likert scale ranging from 1 (strongly disagree) to 5 (strongly agree), with higher scores indicating higher levels of stress within each domain.

The Arabic adaptation of the DSS-A was translated and culturally validated following standard cross-cultural adaptation procedures and has been empirically tested among Arabic-speaking university students (Krägeloh et al., 2023). This adaptation preserved the factorial integrity of the original structure and demonstrated satisfactory psychometric properties, including internal consistency (Cronbach’s α ranging from 0.81 to 0.91 across subscales) and evidence of construct validity in Arab contexts. In the current study, the three selected subscales demonstrated satisfactory internal consistency (Cronbach’s α = 0.78; 0.75 and 0.76), supporting their reliability as measures of digital stress.

### Translation and cultural adaptation

The initial Arabic version of the Digital Life Balance (DLB) Scale was prepared using a forward direct translation approach from English to Arabic, ensuring conceptual and cultural accuracy. The translated version was reviewed by a panel of five experts in psychometrics, Arabic and English languages, and positive psychology.

A Delphi technique was employed to achieve consensus on the wording of the items; the experts’ agreement rate in the first round was 70%. Afterward, revisions were introduced to improve clarity and cultural relevance. In the second round, the agreement rate increased to 85%, allowing the revised version to be approved for preliminary testing.

Finally, a back-translation into English was subsequently conducted by three independent reviewers experienced in academic translation. The agreement rate regarding the accuracy and conceptual equivalence of the back-translated version with the original scale was 90%, supporting the adoption of the final Arabic version for field testing. The final version of the scale is provided in Supplementary Appendix S1.

### Procedure

Data collection was conducted in October 2025 using online survey methods to ensure broad accessibility across the participating countries. Two platforms were utilized for data administration. In Saudi Arabia, the second author distributed the survey link through Qualtrics, following standardized procedures for online research. In Yemen and Egypt, the survey was administered via Google Forms and disseminated through university student groups on Facebook and WhatsApp, in coordination with academic collaborators and student representatives in those countries.

Before accessing the survey, participants were presented with an electronic informed consent form that outlined the purpose of the study, emphasized the voluntary nature of participation, and assured anonymity, confidentiality, and the right to withdraw at any point without penalty. Only individuals who provided explicit consent were allowed to proceed to the survey.

The survey included three main sections: (a) demographic information (e.g., age, gender, and academic level); (b) the Arabic version of the Digital Life Balance Scale (DLBS); and (c) the Arabic version of the Digital Stress Scale (DSS-A), limited to three subscales—Fear of Missing Out, Connection Overload, and Online Vigilance.

To ensure data quality, several screening procedures were applied. Duplicate entries identified by matching IP addresses were removed. Submissions with unusually short completion times, indicating potential inattentive responses, were excluded. The dataset was also screened for incomplete responses and statistical outliers. After these quality checks, a total of 769 valid responses were retained for analysis.

Ethical approval for the study was obtained from the Institutional Review Board (IRB) of Princess Nourah bint Abdulrahman University (IRB Log Number: 25-0587; IRB Registration Number with KACST, KSA: HAP-01-R-059). The study was reviewed under expedited status, as it involved minimal risk to participants. Verbal authorization to conduct the study was obtained from the Faculty of Education at Thamar University in Yemen and from Mansoura University in Egypt.

All procedures adhered to the ethical principles for research involving human participants outlined in the Declaration of Helsinki and other internationally accepted ethical guidelines. Participation was voluntary, informed consent was obtained electronically, and the survey was administered anonymously. Participants were assured of confidentiality and of their right to withdraw from the study at any stage without penalty.

### Data analysis

Data analyses were conducted using IBM SPSS Statistics (version 31) for descriptive statistics and classical reliability estimates (Cronbach’s α and corrected item–total correlations). Confirmatory factor analysis (CFA), composite reliability (CR), and average variance extracted (AVE) were conducted in R (version 4.4.0) using the lavaan package with the WLSMV estimator, which is appropriate for ordinal Likert-type data (Li, 2021; Savalei, 2020). This estimator is recommended over maximum likelihood when data exhibit non-normality or when using ordered categorical indicators.

Preliminary analyses included the examination of descriptive statistics—means, standard deviations, skewness, and kurtosis—to assess the distributional properties of the study variables. Missing data were minimal (<5%) and were treated using the expectation–maximization (EM) method. All DLBS items demonstrated acceptable skewness (±2) and kurtosis (±7) values, indicating no substantial deviations from univariate normality.

The linguistic and cultural equivalence of the Arabic version of the Digital Life Balance Scale (DLBS) was evaluated through a two-round Delphi technique involving five experts in psychometrics, language, and positive psychology. Agreement percentages across rounds were summarized to ensure conceptual and linguistic accuracy.

The construct validity of the Arabic DLBS was assessed using Confirmatory Factor Analysis (CFA) to test the hypothesized one-factor model. Model fit was evaluated using the Comparative Fit Index (CFI), Tucker–Lewis Index (TLI), Root Mean Square Error of Approximation (RMSEA), and Standardized Root Mean Square Residual (SRMR). Interpretation of these indices followed established recommendations (e.g., Hu and Bentler, 1999; Kline, 2016).

Measurement invariance across countries (Saudi Arabia, Yemen, and Egypt) and gender (male vs. female) was tested using multi-group CFA with the WLSMV estimator. We followed a sequential approach comparing: (1) configural invariance (same factor structure), (2) metric invariance (equal factor loadings), (3) scalar invariance (equal item intercepts), and (4) strict invariance (equal residual variances). Invariance was evaluated using changes in CFI (ΔCFI ≤ 0.01) and RMSEA (ΔRMSEA ≤ 0.015) as primary criteria (Chen, 2007; Cheung and Rensvold, 2002).

Convergent validity was examined by computing Pearson’s correlation coefficients (r) between total DLBS scores and the three selected subscales of the Arabic version of the Digital Stress Scale (DSS-A): Fear of Missing Out (FoMO), Connection Overload, and Online Vigilance. Significant negative correlations were hypothesized, consistent with the theoretical expectation that higher digital life balance would be associated with lower digital stress.

In addition, Convergent validity at the latent-factor level was evaluated using standardized factor loadings and the Average Variance Extracted (AVE). Internal consistency was assessed using Cronbach’s alpha (α), McDonald’s omega (ω), and Composite Reliability (CR), with values of 0.70 or higher considered acceptable indicators of reliability.

Item-level analysis was conducted using two complementary psychometric approaches. Within the CFA framework, standardized factor loadings and squared multiple correlations (R^2^) were examined to assess the extent to which each item was explained by the latent construct. In parallel, corrected item–total correlations were computed following classical test theory procedures, reflecting the correlation between each item and the total scale score after excluding the item itself. This approach ensured a clear distinction between CFA-based indicators of item representation and CTT-based indicators of item discrimination.

All statistical tests were two-tailed, and a significance level of *p* < 0.05 was applied for hypothesis testing.

## Results

Preliminary normality checks showed that all DLBS items demonstrated acceptable skewness and kurtosis values within recommended ranges.

### Linguistic and cultural equivalence

The equivalence of the Arabic translation of the Digital Life Balance Scale (DLBS) was evaluated using a two-round Delphi procedure with five experts specializing in psychometrics, Arabic and English language studies, and positive psychology. Experts assessed each item for conceptual clarity, linguistic accuracy, and cultural appropriateness.

During the first round, the overall agreement rate among experts was 70%, which did not meet the predefined acceptance threshold of ≥80%. The primary feedback focused on improving wording clarity, simplifying sentence structure, and ensuring that expressions reflected both the original construct and the local context. See Tables 1, 2 for more details.

**Table 1:** Expert agreement on the Arabic DLBS items across delphi rounds.

Item	Key issue in Round 1	Agreement rate (%)—Round 1	Modification made	Agreement rate (%)—Round 2
1	Clarity of item wording	72%	Revised wording for clarity	88%
2	Conceptual distinction between online and offline domains	68%	Adjusted phrasing for clarity	84%
3	Readability of sentence structure	70%	Simplified statement for comprehension	86%
4	Consistency of terminology across items	69%	Unified wording for a balance between domains	83%
All	–	70%	–	85%

**Table 2:** Summary of experts’ qualitative feedback and revisions.

Item	Round	Main feedback category	Expert comment (summary)	Action taken
1	1	Wording clarity	Improve clarity of wording for better comprehension	Wording revised
2	1	Conceptual clarity	Refine the expression to enhance the conceptual distinction between domains	Phrasing adjusted
3	1	Readability	Simplify the sentence for ease of understanding by students	Sentence simplified
4	1	Terminology consistency	Ensure consistent use of terms across items	Terminology harmonized
All	2	Final confirmation	Experts confirmed conceptual and cultural equivalence	No further changes required

Following these recommendations, the items were revised accordingly and re-evaluated in the second round. The overall agreement rate increased to 85%, surpassing the acceptance criterion. Experts confirmed that the revised Arabic items retained the original conceptual meaning while being linguistically and culturally appropriate for Arabic-speaking university students.

These findings demonstrate that the Arabic version of the DLBS achieved satisfactory linguistic and cultural equivalence with the original English version, thereby supporting H1.

### Confirmatory factor analysis

A confirmatory factor analysis (CFA) was conducted using the lavaan package in R with the WLSMV estimator to examine the construct validity of the Arabic version of the Digital Life Balance Scale (DLBS). The hypothesized one-factor model consisted of four observed indicators (DLB1–DLB4) loading on a single latent construct representing Digital Life Balance.

All standardized factor loadings were statistically significant and exceeded recommended thresholds (see Table 3).

**Table 3:** Standardized factor loadings and squared multiple correlations for the Arabic DLBS.

Item	Standardized loading	*p*-value	*R*^2^
DLB1	0.68	<0.001	0.46
DLB2	0.68	<0.001	0.46
DLB3	0.71	<0.001	0.50
DLB4	0.62	<0.001	0.39

**Figure 1:**
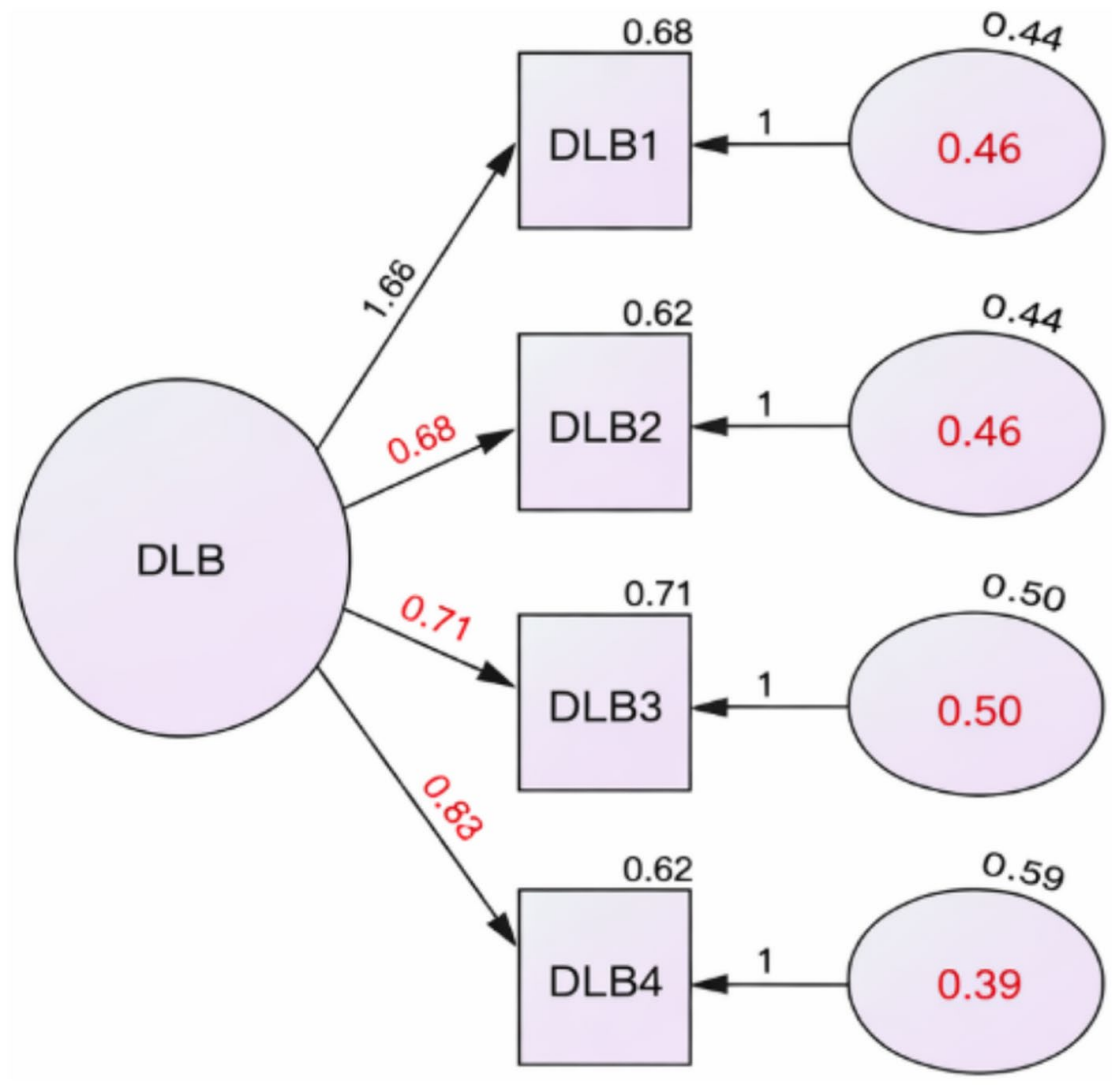
Confirmatory factor analysis model of the Arabic DLB Scale showing standardized factor loadings for all items. The standardized loadings presented in the figure correspond exactly to those reported in Table 3. All factor loadings were statistically significant (*p* < 0.001).

All items demonstrated acceptable factor loadings, suggesting that each contributed meaningfully to the latent construct of Digital Life Balance. The latent factor accounted for 39–50% of the variance in the observed indicators. See Figure 1 for more details. Consistent with these results, the Average Variance Extracted (AVE) for the DLBS factor was 0.45, indicating that the latent construct accounted, on average, for 45% of the variance in its indicators.

Model fit was evaluated using multiple indices in accordance with established guidelines (Hu and Bentler, 1999). As shown in Table 4, the one-factor model demonstrated good to excellent fit to the data: χ^2^(2) = 8.76, *p* = 0.012; CFI = 0.994; TLI = 0.982; RMSEA = 0.065 (90% CI = 0.028–0.109, PCLOSE = 0.24); SRMR = 0.015. The χ^2^/df ratio of 4.38, while above the conventional threshold of 3, is considered acceptable given the sensitivity of χ^2^ to large sample sizes (*N* = 769) and the excellent values of other fit indices (Kline, 2016). The RMSEA value below 0.08 and CFI/TLI values above 0.95 indicate satisfactory model fit.

**Table 4:** Goodness-of-fit indices for the one-factor model of the Arabic DLBS.

Fit index	Value	Recommended threshold	Evaluation
χ^2^ (df = 2)	8.76	–	–
χ^2^/df	4.38	≤5 acceptable	Acceptable
*p*-value	0.012	>0.05 desirable	Expected with large N
CFI	0.994	≥0.95	Excellent
TLI	0.982	≥0.95	Excellent
RMSEA	0.065	≤0.08	Good
RMSEA 90% CI	0.028–0.109	–	–
PCLOSE	0.240	>0.05	Good
SRMR	0.015	≤0.08	Excellent

These results support the one-dimensionality of the Arabic DLBS and confirm that the four observed indicators reliably reflect a single latent construct representing digital life balance. The modification indices suggested no substantial model improvement was necessary, indicating a well-fitting and parsimonious model. Overall, the CFA findings provide empirical support for the construct validity of the Arabic version of the DLBS.

### Measurement invariance across countries and gender

Measurement invariance of the Arabic DLBS was tested across the three countries (Saudi Arabia, Yemen, Egypt) and gender (male vs. female) using multi-group CFA with the WLSMV estimator. As shown in Table 5, configural invariance was established for both grouping variables, indicating that the one-factor structure holds across groups. Metric invariance was supported across countries (ΔCFI = 0.003, ΔRMSEA = 0.002) and gender (ΔCFI = 0.002, ΔRMSEA = 0.001), suggesting equivalent factor loadings. Scalar invariance was also supported across countries (ΔCFI = 0.006, ΔRMSEA = 0.003) and gender (ΔCFI = 0.005, ΔRMSEA = 0.002), indicating that item intercepts were equivalent. These results demonstrate that the Arabic DLBS measures the same construct in the same metric across the examined groups, allowing for meaningful comparisons of latent means and relationships with other variables.

**Table 5:** Measurement invariance testing of the Arabic DLBS across countries and gender.

Model	χ^2^ (df)	CFI	RMSEA [90% CI]	SRMR	ΔCFI	ΔRMSEA	Invariance decision
Across countries							
Configural	45.21 (6)	0.985	0.074 [0.055, 0.095]	0.025	–	–	Supported
Metric	52.34 (14)	0.982	0.072 [0.053, 0.092]	0.028	0.003	0.002	Supported
Scalar	68.45 (22)	0.979	0.069 [0.052, 0.087]	0.031	0.006	0.003	Supported
Across gender							
Configural	28.76 (2)	0.988	0.081 [0.058, 0.106]	0.019	–	–	Supported
Metric	33.45 (5)	0.986	0.080 [0.057, 0.104]	0.022	0.002	0.001	Supported
Scalar	42.18 (8)	0.983	0.079 [0.056, 0.103]	0.025	0.005	0.002	Supported

### Convergent validity with the digital stress scale

The convergent validity of the Arabic Digital Life Balance Scale (DLBS) was evaluated by examining its associations with the three selected subscales of the Arabic Digital Stress Scale (DSS-A): Fear of Missing Out (FoMO), Connection Overload, and Online Vigilance. These dimensions represent core indicators of digital stress that are theoretically expected to be inversely related to digital life balance.

As shown in Table 6, the total DLBS score demonstrated significant negative correlations with two of the three digital stress dimensions—FoMO (*r* = −0.10, *p* = 0.006) and Connection Overload (*r* = −0.15, *p* < 0.001)—while its association with Online Vigilance was negative but nonsignificant (*r* = −0.07, *p* = 0.060). The magnitude of these relationships was small, consistent with theoretical expectations.

**Table 6:** Correlations between the Arabic (DLBS) and the three dimensions of the Arabic (DSS-A).

DSS-A dimension	*r*	*p*-value	Interpretation
Fear of missing out (FoMO)	−0.100**	0.006	Small negative correlation
Connection overload	−0.153**	<0.001	Small negative correlation
Online vigilance	−0.068	0.060	Negative, nonsignificant

*N* = 769. **p < 0.01 (two-tailed). Negative correlations indicate that higher digital life balance is associated with lower levels of digital stress.

The observed pattern of correlations supports the hypothesized inverse relationship between digital balance and digital stress. Specifically, individuals reporting higher digital life balance tended to experience less fear of missing out and lower connection overload, suggesting that maintaining equilibrium between online and offline life is associated with reduced susceptibility to key forms of digital strain. The nonsignificant relationship with online vigilance may reflect that this behavior involves both adaptive (e.g., awareness and monitoring) and maladaptive (e.g., compulsive checking) elements, which can dilute the overall association with digital balance.

These findings provide preliminary and limited evidence for the convergent validity of the Arabic DLBS, given the small magnitude of the observed correlations.

### Internal consistency and composite reliability

As shown in Table 7, the DLBS demonstrated satisfactory internal consistency and composite reliability.

**Table 7:** Internal consistency, composite reliability, and average variance extracted.

Reliability index	Criterion	Obtained value	Interpretation
Cronbach’s α	≥0.70	0.87	Acceptable
McDonald’s ω	≥0.70	0.80	Acceptable
Composite reliability (CR)	≥0.70	0.89	Acceptable
Average variance extracted (AVE)	≥0.50	0.45	Near acceptable

Finally, as presented in Table 8, corrected item–total correlations computed using classical test theory procedures ranged from 0.53 to 0.60, exceeding the commonly recommended minimum threshold of 0.30 and supporting adequate item discrimination.

**Table 8:** Corrected item–total correlations (CTT).

Item	Corrected item–total correlation
DLB1	0.567
DLB2	0.578
DLB3	0.596
DLB4	0.532

Overall, the results indicate that the Arabic DLBS demonstrates strong internal consistency and a coherent one-factor structure within the examined Arabic-speaking university student sample, with convergent validity evidence that is partial.

## Discussion

The purpose of this study was to translate, culturally adapt, and validate the Arabic version of the Digital Life Balance Scale (DLBS) among Arab university students. The findings supported the proposed hypotheses, providing strong evidence for the linguistic, cultural, and psychometric validity of the Arabic DLBS.

The results of the Delphi evaluation confirmed the first hypothesis (H1), demonstrating satisfactory linguistic and cultural equivalence between the Arabic and the original English versions. The iterative expert review process, which achieved an agreement rate of 85% in the second round, indicated that the translation maintained both conceptual integrity and cultural relevance. These findings are consistent with established recommendations in cross-cultural instrument adaptation, emphasizing the importance of expert consensus to ensure semantic and conceptual accuracy (Beaton et al., 2000; Sousa and Rojjanasrirat, 2011). Similar adaptation procedures in other linguistic validations of the DLBS (Soysal et al., 2024; Lima-Costa et al., 2024) have also highlighted the necessity of expert validation to secure cross-cultural comparability.

Confirmatory Factor Analysis results supported the second hypothesis (H2), showing an acceptable model fit for the one-factor structure. All items exhibited high standardized loadings, confirming the unidimensional nature of the construct. This outcome aligns with previous validation studies of the DLBS in European and Latin American contexts (Duradoni et al., 2022; Lima-Costa et al., 2024), where a single latent factor representing Digital Life Balance was consistently supported. The findings indicate that the Arabic version of the Digital Life Balance Scale demonstrates a coherent one-factor structure within the studied Arabic-speaking university student sample.

The measurement invariance testing across countries and gender represents an important psychometric contribution within the studied Arabic-speaking university student sample. The establishment of configural, metric, and scalar invariance indicates that the Arabic DLBS measures the same construct with equivalent factor loadings and item intercepts across Saudi, Yemeni, and Egyptian students, as well as across male and female respondents. This psychometric evidence supports the use of the scale for meaningful cross-group comparisons within Arab contexts, addressing a notable gap in previous cross-cultural adaptations of digital well-being measures.

Furthermore, the use of the WLSMV estimator in CFA represents a methodological improvement over traditional maximum likelihood estimation, particularly given the ordered categorical nature of Likert-scale data. This approach provides more robust parameter estimates and fit indices, enhancing the validity of our structural analyses. The good model fit achieved with this estimator, despite the elevated χ^2^/df ratio—a common occurrence with large samples—strengthens confidence in the one-factor structure of the Arabic DLBS.

The third hypothesis (H3), concerning convergent validity, was partially supported. Significant negative correlations were observed between the DLBS and two of the three subdimensions of the Arabic Digital Stress Scale (DSS-A)—Fear of Missing Out and Connection Overload—while the correlation with Online Vigilance was negative but nonsignificant. The significant correlations with FoMO and Connection Overload were small in magnitude, which aligns with the limited conceptual overlap expected between digital life balance and these specific dimensions of digital stress. These results are consistent with theoretical expectations that individuals with higher levels of digital life balance experience lower digital stress related to social connectivity and communication overload (Duradoni et al., 2024; Malas et al., 2025). The nonsignificant association with online vigilance may reflect the dual nature of this construct, which includes both adaptive behaviors (e.g., awareness and digital monitoring) and maladaptive tendencies (e.g., compulsive checking), potentially attenuating its overall relationship with digital balance.

The fourth hypothesis (H4) was confirmed, as the Arabic DLBS demonstrated high internal consistency and strong composite reliability, alongside an Average Variance Extracted of 0.45. Although the AVE was slightly below the conventional 0.50 benchmark, the uniformly high factor loadings and composite reliability indicate strong internal consistency, while evidence for convergent validity at the latent level should be interpreted with caution. These results are comparable to those reported in other cross-cultural validations (Duradoni et al., 2022; Soysal et al., 2024), reinforcing the reliability and coherence of the Arabic version as a psychometric instrument. Collectively, these findings indicate that the Arabic DLBS is a reliable and valid tool for assessing individuals’ perceived ability to maintain a balanced integration between online and offline domains.

The findings extend the theoretical framework of Digital Life Balance to the Arab cultural setting, highlighting that balanced digital engagement is associated with reduced stress and improved psychological well-being. The replication of the one-factor structure and the observed pattern of correlations with selected digital stress dimensions provide empirical support for the construct’s applicability within the examined sample.

The nonsignificant association between DLBS and Online Vigilance warrants consideration. Although a negative trend was observed, the absence of statistical significance may reflect conceptual distinctions between the two constructs in Arabic-speaking contexts. Online Vigilance emphasizes constant monitoring and socio-digital alertness, whereas digital life balance captures the regulation of digital engagement. The item content of the Online Vigilance scale may tap into behaviors that are culturally normative among university students and therefore not perceived as dysregulating. Age homogeneity within the sample may also have limited variability in vigilance-related behaviors. Finally, it remains plausible that Online Vigilance is not strongly aligned with digital balance in Arab populations, suggesting the need for further investigation of the construct’s cultural applicability.

Despite the study’s methodological rigor, several limitations should be acknowledged. First, the sample was limited to university students from three Arab countries, which may restrict the generalizability of the results to other demographic or occupational groups. Second, although participants were drawn from three countries, the sampling procedure followed a convenience-based approach and did not yield balanced or representative national subsamples. Although measurement invariance across the three countries was supported at the configural, metric, and scalar levels, the convenience sampling strategy and unequal group sizes may limit the generalizability of cross-country comparisons. Accordingly, the present findings should be interpreted primarily as evidence drawn from a pooled Arabic-speaking university student sample, while the robustness of cross-national equivalence would benefit from replication using stratified and more balanced multi-country samples. Future research employing stratified or population-based sampling designs would be better positioned to evaluate measurement invariance across Arab cultural contexts. Third, the cross-sectional design precludes causal inferences between digital balance and digital stress. Fourth, the reliance on self-report measures introduces the possibility of social desirability bias. Fifth, the absence of test–retest reliability analysis limits conclusions about the scale’s temporal stability. Sixth, only three DSS-A subscales were used rather than the full five-subscale structure. Although these subscales are theoretically the closest to digital life balance, excluding the remaining dimensions narrows the scope of the convergent validity assessment. Future research may therefore benefit from incorporating the full DSS-A to provide a more comprehensive evaluation of construct convergence. Seventh, while measurement invariance was successfully established across the three included countries and gender, we did not test invariance across age groups due to limited age variability in our university student sample. Future research should examine whether the scale functions equivalently across different age cohorts within Arab populations. Finally, while participants represented three countries, further validation across a wider range of Arabic-speaking regions is needed to confirm measurement invariance across subcultures.

Future research should aim to validate the Arabic DLBS in more diverse demographic and occupational groups, including adolescents, professionals, and older adults, to broaden its applicability. Longitudinal designs are recommended to investigate causal relationships between digital balance, digital stress, and well-being. Studies should also examine measurement invariance across different Arab countries to confirm the scale’s cross-regional validity. Additionally, incorporating qualitative approaches such as cognitive interviews or focus groups could offer deeper insights into culturally specific interpretations of digital balance. Finally, test–retest reliability and predictive validity analyses are recommended to further strengthen the psychometric evidence base of the Arabic DLBS.

## Conclusion

This study translated, culturally adapted, and psychometrically evaluated the Arabic version of the Digital Life Balance Scale (DLBS) among Arab university students. The findings demonstrated satisfactory linguistic and cultural equivalence, a clear one-factor structure, and strong internal consistency and composite reliability. Convergent validity was examined using three subscales of the Arabic Digital Stress Scale (DSS-A). Digital life balance showed small, significant negative associations with Fear of Missing Out and Connection Overload, whereas its association with Online Vigilance was negative but nonsignificant, indicating partial support for convergent validity.

The confirmatory factor analysis supported the conceptual coherence of Digital Life Balance as a unidimensional construct within the examined Arabic-speaking university student sample. Overall, the results indicate that higher levels of digital life balance are associated with lower levels of certain digital stressors, particularly those related to social pressure and communication overload.

By establishing the reliability, factor structure, and partial convergent validity of the Arabic DLBS, the present study contributes to the literature on digital behavior and well-being in Arabic-speaking contexts. The validated scale provides a culturally relevant and psychometrically sound tool for assessing the balance between online and offline life and offers a foundation for future empirical research and applied work in Arab populations.

Accordingly, the findings should be interpreted as evidence derived from a pooled Arabic-speaking university student sample rather than as representative of specific national contexts.

## Data Availability

The raw data supporting the conclusions of this article will be made available by the authors, without undue reservation.
