# Supplementary material for: Translation, cultural adaptation, and psychometric validation of the Arabic version of the Digital Life Balance Scale in an Arabic-speaking university student sample
Source: Front Psychol. 2026 Feb 12;17:1741166. doi: 10.3389/fpsyg.2026.1741166 (PMC12936027; doi:10.3389/fpsyg.2026.1741166)
Supplement: Supplementary file 1 [file Data_Sheet_1.pdf]

Appendix 1
The final version of the scale

الجدول التالي يتضمن البنود الأصلية باللغة الإنجليزية، والترجمة العربية المقترحة، والصورة النهائية للمقياس.

البند الأصلي	الترجمة العربية المقترحة	الصورة النهائية للمقياس
I currently have a good balance between the time I spend online and the time I have available for offline activities.	أشعر حالياً بأن لدي توازناً جيداً بين الوقت الذي أقضيه على الإنترنت والوقت المتاح لدي للأنشطة غير المتصلة بالإنترنت	أشعر حالياً بأن لدي توازناً جيداً بين الوقت الذي أقضيه على الإنترنت والوقت المخصص للأنشطة الواقعية
I have difficulty balancing my online and offline activities. [R]	أجد صعوبة في تحقيق توازن بين أنشطتي على الإنترنت وأنشطتي خارج الإنترنت (بند معكوس)	أواجه صعوبة في الموازنة بين أنشطتي على الإنترنت وأنشطتي الواقعية (بند معكوس)
I feel that the balance between my online and offline activities is currently about right.	أشعر أن التوازن بين أنشطتي على الإنترنت وأنشطتي خارج الإنترنت مناسب حالياً	أشعر أن التوازن بين أنشطتي على الإنترنت وأنشطتي الواقعية مناسب حالياً
Overall, I believe that my online and offline life are balanced.	بصورة عامة، أعتقد أن حياتي على الإنترنت وخارج الإنترنت متوازنتان	بصورة عامة، أعتقد أن أنشطتي على الإنترنت وأنشطتي الواقعية متوازنة
